# Using Social Media Mining and PLS-SEM to Examine the Causal Relationship between Public Environmental Concerns and Adaptation Strategies

**DOI:** 10.3390/ijerph18105270

**Published:** 2021-05-15

**Authors:** Chia-Lee Yang, Chi-Yo Huang, Yi-Hao Hsiao

**Affiliations:** 1 National Center for High-Performance Computing, Hsinchu City 300, Taiwan; joy.yang@nchc.org.tw (C.-L.Y.); ihow@nchc.narl.org.tw (Y.-H.H.); 2 Program of Technology Management, Department of Industrial Education, National Taiwan Normal University, Taipei 106, Taiwan

**Keywords:** environmental concerns, air pollution, social media, value-belief-norm model, partial least squares technique of structural equation modelling (PLS-SEM), gender difference

## Abstract

With growing scientific evidence showing the harmful impact of air pollution on the environment and individuals’ health in modern societies, public concern about air pollution has become a central focus of the development of air pollution prevention policy. Past research has shown that social media is a useful tool for collecting data about public opinion and conducting analysis of air pollution. In contrast to statistical sampling based on survey approaches, data retrieved from social media can provide direct information about behavior and capture long-term data being generated by the public. However, there is a lack of studies on how to mine social media to gain valuable insights into the public’s pro-environmental behavior. Therefore, research is needed to integrate information retrieved from social media sites into an established theoretical framework on environmental behaviors. Thus, the aim of this paper is to construct a theoretical model by integrating social media mining into a value-belief-norm model of public concerns about air pollution. We propose a hybrid method that integrates text mining, topic modeling, hierarchical cluster analysis, and partial least squares structural equation modelling (PLS-SEM). We retrieved data regarding public concerns about air pollution from social media sites. We classified the topics using hierarchical cluster analysis and interpreted the results in terms of the value-belief-norm theoretical framework, which encompasses egoistic concerns, altruistic concerns, biospheric concerns, and adaptation strategies regarding air pollution. Then, we used PLS-SEM to confirm the causal relationships and the effects of mediation. An empirical study based on the concerns of Taiwanese social media users about air pollution was used to demonstrate the feasibility of the proposed framework in general and to examine gender differences in particular. Based on the results of the empirical studies, we confirmed the robust effects of egoistic, altruistic, and biospheric concerns of public impact on adaptation strategies. Additionally, we found that gender differences can moderate the causal relationship between egoistic concerns, altruistic concerns, and adaptation strategies. These results demonstrate the effectiveness of enhancing perceptions of air pollution and environmental sustainability by the public. The results of the analysis can serve as a basis for environmental policy and environmental education strategies.

## 1. Introduction

Air pollution has become a major problem in modern societies due to its significant impacts on human health, the climate, and the ecosystem in general [1]. Epidemiological research has shown that there is a relationship between air pollutants and risk of diseases such as type 2 diabetes mellitus [2], rheumatoid arthritis [3], Parkinson’s disease [4], as well as the incidence of chronic obstructive pulmonary disease (COPD) and asthma [5]. An estimated seven million deaths are associated with air pollution each year according to the World Health Organization (WHO), much greater than the number who die from acquired immune deficiency syndrome (AIDS), tuberculosis, and traffic accidents combined [6]. The economic burden associated with health damage from ambient air pollution internationally is colossal, estimated to be $5.7 trillion, or 4.8% of global gross domestic product (GDP) annually, according to a 2020 World Bank report [7]. 

In response to air pollution, many countries have developed action plans for reducing air pollution through air quality monitoring and warning systems, the management of toxic or otherwise potentially harmful chemicals, and energy efficiency and clean energy, among other measures. These action plans often aim to modify individual behavior that reduces air pollution, such as encouraging people to consume green and clean products [8]. Previous studies have shown that public environmental concerns are the main function that is undertaken with the intention to change the environment [9,10]. People who are more likely to participate in activities to reduce air pollution are those who have been concerned about and are aware of problems related to air pollution [11]. Thus, public awareness and support are important for environmental policies designed to resolve air pollution issues.

Public perception of the environment is both a social and an environmental issue. Recently, numerous scholars have investigated the relationships among public perceptions and air pollution [12]. The theories of reasoned action [13] and planned behavior [14], the norm-activation model [15], and value-belief-norm theory [10] were adopted to explain the relationship between individual beliefs and their pro-environmental behavior, every probable action intended for protecting the environment. Most research, e.g., [16,17,18,19], has applied these theories to analysis of the relationship between attitudes and behaviors among survey respondents. 

Although statistical sampling methods may have good data quality and provide powerful support for a theoretical framework, the sampling data sometimes involves biased selection, and the results may not be generalizable to the entire population. In addition, stratified sampling captures a wide variety of participant characteristics, but may not be representative of the underlying population [20]. Mining social media may provide an opportunity to survey the population directly and retrieve public opinions in real time and on a massive scale [21]. 

In comparison with data gathered through approaches based on statistical sampling, data retrieved from social media can be used to monitor or directly track information being generated by the public in real time [22]. It also allows for the tracking of individual interactions with society on environmental issues. Recent studies have used social media to monitor trends regarding air quality as well as related public attitudes and responses [21,23]. However, data from social media is relatively messy and in an unstructured, raw format. To the best of our knowledge, no study thus far has mined social media data content on air pollution and interpreted this data through environmental concern theories. Investigating the causal relationship between environmental concerns and psychological adaptation toward air pollution is important for incorporating environmental values retrieved from social media and behavior in a causal chain.

Thus, a theoretical framework is needed to integrate information retrieved from social media sites into an established theoretic framework of environmental behaviors. Therefore, in this paper, we aim to construct a theoretical model by integration of social media mining into a value-belief-norm theory model of the public’s concerns about air pollution. Additionally, there is growing epidemiologic evidence of differing associations between air pollution and respiratory health for men and women [24,25]. Women are also more aware of environmental hazards that often motivate social action than men because significant differences exist between the genders in terms of knowledge, awareness, and attitudes [26,27]. Considerations of gender differences in terms of air-quality issues provide new insights for researchers and policy makers. Thus, we also integrate gender differences into the value-belief-norm theory model as a moderating variable. 

To retrieve information from social media and confirm the value-belief-norm model, we propose a hybrid method that integrates text mining, topic modeling, hierarchical cluster analysis, and partial least squares structural equation modelling (PLS-SEM). We retrieved data regarding public concerns about air pollution from social media sites. We classified the topics using hierarchical cluster analysis and interpreted the results in terms of the value-belief-norm theoretical framework, which encompasses egoistic concerns, altruistic concerns, biospheric concerns, and adaptation strategies regarding air pollution. Then, we used PLS-SEM to confirm the causal relationships and the effects of mediation. An empirical study based on the concerns of Taiwanese social media users about air pollution was used to demonstrate the feasibility of the proposed framework in general and to examine gender differences in particular.

## 2. Literature Review

Environmental concern is the degree to which people are aware of environmental problems and support efforts to solve these problems and/or indicate a willingness to contribute solutions to such problems [28]. Environmental concern could be the beliefs, affects, and behavioral intentions a person holds regarding activities or issues related to the environment [29]. There is wider recognition that environmental concerns are the main drivers of the intention to change the environment—that is, to engage in pro-environmental behavior [9]. The value-belief-norm theory was first established by Stern et al. [30] to explain the influence of human values on behavior in an environmentalist context. Thus, in Section 2.1, we first review environmental concerns and the value-belief-norm theory. Public concern and support are one of the most important resources of social movements [30]. There is a consensus in the literature regarding the strong linkages between public perceptions of air pollution and emotional and behavioral responses to air pollution [12,31]. Thus, following the review of the value-belief-norm theory, we review public perceptions of air pollution. In the past years, social media has emerged as one of the most important sources for monitoring and detecting environmental problems in general and air pollution problems in particular. In Section 2.3, we review recent advances in adopting social media for the detection of air pollution. Then, we review recent research and governmental efforts to control air pollution in Taiwan, the target of the empirical study. Finally, Section 2.5 will define the hypotheses based on the path model linking environmental concerns—which include egoistic, altruistic, and biospheric concerns based on the value-belief-norm theory—to adaptation for air pollution.

### 2.1. Environmental Concerns and the Value-Belief-Norm Theory

Previous studies have used the theories of reasoned action [13] and planned behavior [14], the norm-activation model [15], and the value-belief-norm theory [10] to explain pro-environmental behavior [12]. The norm-activation model being proposed by Shwartz et al. [32] was developed to describe factors that influence helping behavior that is performed for altruistic reasons. According to the norm-activation model, altruistic helping behaviors occur in response to personal moral norms, awareness of consequences, and the ascription of responsibility. Pro-environmental behaviors can be conceptualized as being altruistic because those who perform them are doing them to protect the natural environment and society. 

Stern et al. [10] extended the norm-activation model by proposing the value–belief–norm theory. A value is a guiding principle for any behavior based on desirable trans-situational goals and aligned with the environmentalism and develops increasing beliefs that end up in the development of a pro-environmental personal norm. The value-belief-norm theory proposed that the individuals who accept a movement’s basic values believe that valued objects are threatened. Due to this, these individuals believe that their actions can help restore those values and experience individual norms for a pro-movement action. The action creates a predisposition to contributing and/or giving support at different levels. Instead of the awareness of consequences, the value-belief-norm theory incorporated beliefs about adverse consequences and posited relationships between values, beliefs, norms, and behaviors in a causal chain. According to the value-belief-norm theory, people’s attitudes about environmental issues are determined by the value that they place on themselves (egoistic value), other people (social-altruistic value), and plants and animals (biospheric values) [10]. The basic components of the value-belief-norm theory are the three value orientations regarding environmental concerns, which include egoistic, altruistic, and biospheric concerns. 

Egoistic concerns can be reflected in concerns about environmental problems at the individual level. These concerns include personal health, financial well-being, quality of life, availability of resources, and so on. Altruistic concerns, also called social-altruistic concerns, focus on people other than oneself, including friends, family, community, future generations, or humanity. People with social-altruistic environmental attitudes care about environmental problems because the problems affect other people. People with biospheric concerns focus on all living things, which include plants, animals, ecosystems, and the biosphere. The value-belief-norm theory has been successfully applied to predicting people’s pro-environmental behavior in a broad range of circumstances. For example, De Dominicis et al. [33] examined the different values and found that these values had different effects on pro-environmental behaviors in the cases of conserving energy, using public transportation, and participating in a beach clean-up event. Imaningsih et al. [34] analyzed the influence of egoistic, altruistic and biospheric values on green functional benefits, green monetary cost, green satisfaction, and green loyalty. They found that egoistic and supply chain values have a positive effect on green loyalty, while altruistic values do not affect green loyalty.

### 2.2. Public Perceptions of Air Pollution

Air pollution is a major global problem that threatens human life and health as well as the environment [35]. Thus, the different kinds of pro-environment actions of reducing air pollution have been studied, such as personal buying behavior, travel behavior, recycling and use of resources, and active participation [36]. Success and effectiveness in controlling air pollution, specifically at the local level, largely depends on social movements. Public concern and support is one of the most important resources of social movements [30]. There is a consensus in the literature regarding the strong linkages between public perceptions of air pollution and emotional and behavioral responses to air pollution [12,31]. 

Unlike professional air pollution evaluations based on scientific data, public perceptions of air pollution appear to be heterogeneous, and complicated by various influential factors and mechanisms. In general, the studies that have been conducted to assess how people perceive air pollution have found that such perceptions depend on local or regional factors. For example, researchers have argued that the public’s perception of air pollution in urban and suburban areas [37] as well as specific area pollution exposure is different [38,39]. In addition, the public perception of air pollution is also socially constructed and related to individuals’ characteristics and physical and social dimensions. Several studies have been conducted to assess correlations of individual difference to concerns regarding air pollution from psychological and sociological perspectives. The researchers argued that the individual factors such as knowledge [31], race [40], and gender [41] can moderate the influence of individual perceptions of air pollution. For example, gender appears to be one of the primary characteristics related to physical and social dimensions. The research demonstrates that women have a higher level of awareness about everyday hazards such as air pollution, which often motivates social action [42,43,44]. Garcia-Vargas et al. [44] found that women constitute the population most vulnerable to climate change and climate variability, and women have important knowledge and skills for orienting the adaptation processes. Although gender is a central organizing principle in social life, gender issues are rarely examined by environment disaster scholars or practitioners. Researchers draw attention to critical limitations and occlusions concerning gender minorities in environment disaster risk reduction policy, which may be a critical focus for future collaborative and applied research [45].

### 2.3. Air Pollution Detection by Using Social Media 

Social media are defined as “web-based services that allow individual, communities, and organizations to collaborate, connect, interact, and build community by enabling them to create, co-create, modify, share, and engage with use-generated content that is easily accessible” [46]. In the past decade, social media have been widely adopted by many internet users. About two-thirds of adults in the developed world and high percentages of users in emerging and developing economies have adopted social media [47]. Social media provide users with tools to represent themselves, expand their interpersonal networks, construct social identities, and maintain or create new social bonds. Social media have become an integral part of everyday life with large economic, political, and societal implications.

As air pollution is a serious environmental problem in many developing countries, obtaining timely and accurate information is an important first step toward control of air pollution. Traditionally, researchers have used questionnaire surveys and interviews to collect data on perceived air quality and people’s displeasure with air pollution [18,19,48]. Recent studies have demonstrated the application of social media for monitoring air quality fluctuation as well as related public attitudes and responses [21,23]. For example, Wang et al. [21] found that social media contains rich detail regarding perceptions, behaviors, and self-reported health effects, which can augment existing air pollution surveillance data, especially the sources of perceptions and health-related data. Hswen et al. [22] proposed that social media may offer a supplemental source of data, and conducted a study in which they used data from Twitter to validate data from established air pollution monitoring stations in a densely populated urban area in London, England.

### 2.4. Air Pollution in Taiwan

Due to its long-lasting detrimental effects, which have led to disease and illness in, for example, the south-east Asia region, air pollution is the greatest environmental risk to human health [6]. Taiwan is facing this problem in the course of seeking equilibrium between environmental protection and economic growth. Some studies have explored the connection between air pollution and disease in Taiwan. For example, Tseng et al. [49] investigated the effects of prevalence changes in tobacco smoking and particulate matter (PM) 2.5 levels on lung cancer by examining 371,084 lung cancer patients in Taiwan. According to the work by Tseng et al. [49], more than 50% of patients with lung cancer had never smoked, and that PM2.5 level changes can affect adenocarcinoma lung cancer incidence and patient survival. Lee et al. [4] investigated the influence of ambient air pollution on Parkinson’s disease by reviewing data of 11,117 patients from the Taiwanese National Health Insurance Research Database and selected 44,468 age- and gender-matched population controls from the longitudinal health insurance database. They found that ambient air pollution exposure, especially from traffic-related pollutants such as nitrogen oxides and carbon monoxide, increases the risk of Parkinson’s disease.

Due to the rising rates of cardiovascular and lung disease in Taiwan, air pollution has attracted great attention from Taiwanese citizens. Reflecting the importance of air quality, the Taiwanese government implemented a strategic plan in 2016 to address air pollution control. Furthermore, the pursuit of better air quality has urged policy-makers to initiate monitoring of air pollution: the government built more than 77 air quality monitoring stations in the country as of 2020, according to the Taiwan Air Quality Monitoring Network (TAQMN) [50].

### 2.5. Hypothesis Development

Based on the literature review presented thus far, we propose a path model linking environmental concerns to adaptation for air pollution. The strategies for adapting to air pollution were proposed to be influenced by three factors—egoistic concerns, altruistic concerns, and biospheric concerns—based on the value-belief-norm theory. The value-belief-norm theory proposed by Stern et al. [30] argues that the three environmental concerns—egoistic, altruistic, and biosphere concerns—impact the development of personal generic beliefs regarding the environment. Helm et al. [51] have had similar findings. De Groot and Steg [52,53] demonstrated that altruistic and biospheric values are highly correlated. Furthermore, for most of the cases, altruistic and egoistic values are correlated [53,54,55] while the correlation between egoistic concerns and biosphere concerns is not significant [53]. Thus, we hypothesize:

**Hypothesis** **1** **(H1).** *Altruistic concerns about air pollution are positively associated with egoistic concerns about air pollution*.

**Hypothesis** **2** **(H2).** *Altruistic concerns about air pollution are positively associated with biosphere concerns about air pollution*.

Adaptation strategies are the plans of adjustment in natural or human systems in response to actual or expected environmental disaster by increasing a system’s ability or reducing its vulnerability [56]. Pro-environmental behaviors are actions that can protect a specific ecosystem or the environment as a whole from the destructive effects of human activities [51]. Egoistic concerns are expressed as functional benefits and emotional benefits [57]. Individuals with egoistic concerns take care of their own welfare. Sarpong et al. [58] showed that egoistic and altruistic environmental concerns were positive drivers of consumer willingness to purchase efficient, water-saving appliances. Aprile and Fiorillo [59] showed that there is a positive correlation between egoistic environmental concerns and water conservation behavior when general environmental issues are perceived as a threat to one’s own welfare. The more strongly people endorse egoistic concerns, the more they are preoccupied with specific local environmental issues that directly affect them, rather than feeling stressed about global issues such as climate change [60]. We believe that people may adopt air pollution adaptation strategies if air pollution problems are perceived to impact their own well-being by increasing the personal costs of environmental degradation. In this context, individuals would adopt air pollution adaptation strategies with the aim of internalizing future personal costs. Based on this argument, we hypothesize:

**Hypothesis** **3** **(H3).** *Egoistic concerns about air pollution are positively associated with air pollution adaptation strategies*.

Several studies have found that altruistic concerns are stronger among people who are engaged in pro-environmental activities [60]. Endorsing altruistic values may lead to perceived environmental stress and coping [51]. As with most prosocial behavior, pro-environmental behavior has inherent characteristics of altruism [61]. Several studies have found that altruistic values are stronger among people who engage in pro-environmental activities [62]. Further research found that altruistic values have a positive impact on consumers’ attitudes and purchase intentions towards eco-friendly packaged products [63]. Thus, we hypothesize:

**Hypothesis** **4** **(H4).** *Altruistic concerns about air pollution are positively associated with air pollution adaptation strategies*.

Several studies have found that biospheric concerns are connected with the pro-environmental behavior intension. Nguyen et al. [64] found biospheric value to encourage active engagement in pro-environmental purchase behavior by enhancing consumers’ attitudes towards environmental protection, their subjective norms and environmental self-identity, and by mitigating their perceived inconvenience associated with eco-friendly products. Kiatkawsin et al. [65] found the biospheric values showed a strong influence on consumers’ likelihood of choosing sustainable products. Imaningsih et al. [34] found the egoistic and biospheric values to have a positive effect on green loyalty. Thus, we hypothesize:

**Hypothesis** **5** **(H5).** *Biosphere concerns about air pollution are positively associated with strategies for adaptation to air pollution*.

Previous studies show that women tended to show higher levels of concern about environmental problems than men [60,66]. Mersha and van Laerhoven [67] proposed that gender differences exist in the barriers to adaptation of responses to climate change in Ethiopia. Vicente-Molina et al. [68] found that women are more altruistically motivated and behave more responsibly towards the environment than men. Liao et al. [48] found that women tend to have a higher knowledge of air pollution than men. Thus, we hypothesize:

**Hypothesis** **6a–e** **(H6a–e).** *Gender has a moderated mediation effect on the relationships among egoistic concerns, altruistic concerns,biosphere concerns, and air pollution adaptation strategies*.

**Figure 1:**
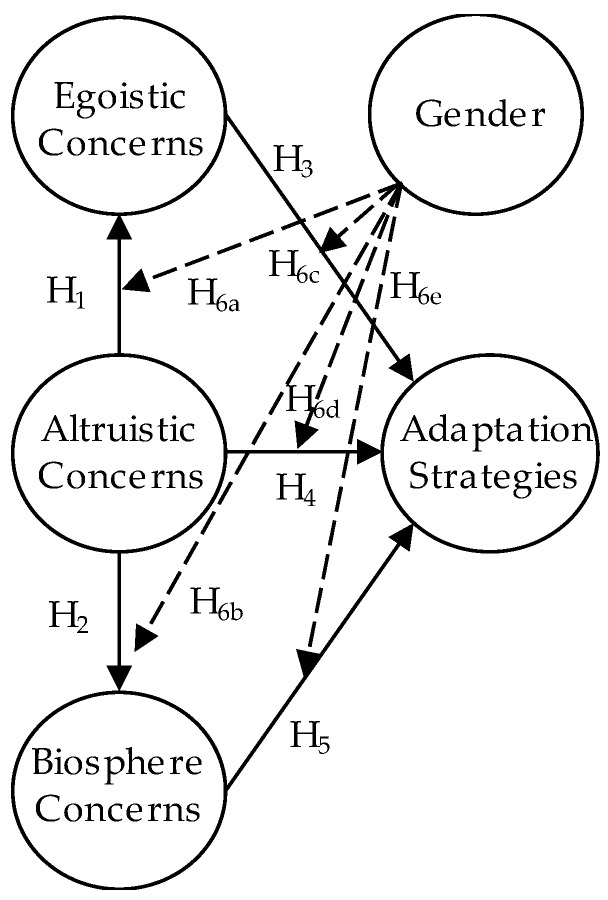
The proposed research model.

The proposed research model is presented in Figure 1.

## 3. Research Method

To retrieve information from social media and confirm the value-belief-norm model, we propose a hybrid method integrating the text mining, topic modeling, hierarchical clustering and the partial least squares structural equation modelling (PLS-SEM). We first used the Dcard (www.dcard.tw; accessed on 20 October 2020), one of the largest social media in Taiwan in which all the messages are tagged by gender, as the data source to collect posts and gender information regarding air pollution. Then, the latent Dirichlet allocation (LDA) technique was used to derive topic models from Dcard. Based on the probability scores obtained through topic modeling, hierarchical cluster analysis was performed to identify the themes of the topic clusters, which includes egoistic concerns, altruistic concerns, biosphere concerns and adaptation strategies regarding air pollution by using hierarchical clustering. After all the probability scores were populated for all the topics, the scores were normalized on a scale of 1 to 5. The resulting matrix is the dataset for PLS-SEM analyses. Finally, the PLS-SEM was introduced to confirm the causal relationships among the topics, and the mediation effects additionally. Accordingly, the theoretical framework consisting of egoistic concerns, altruistic concerns, and adaptation strategies regarding air pollution is confirmed. Additionally, gender differences were considered and adopted as a moderating variable in the theoretical model.

In this study, we used user-generated textual data to generate the dataset for quantitative analysis as well as for generating the dataset for PLS-SEM. The hybrid framework was inspired by previous research. Saga et al. [69] proposed a hybrid framework integrating the LDA and proposed a process of analyzing SEM using LDA. Ray et al. [70] also adopted the LDA and the SEM to explore various values affecting behavioral intention in the context of e-learning services. Both studies used the words as observation variables and the topics as the latent variables for SEM. This paper extends the above method to the multi-topic model, using topics as observation variables and topic clusters as latent variables, avoiding missing too much information due to the diverse information on social media.

**Figure 2:**
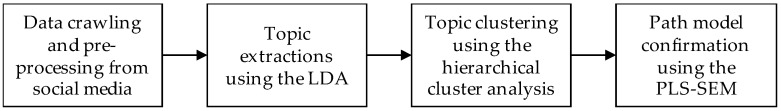
Research Framework.

Below, the methods will be introduced. The three data analytic techniques, namely, topic modelling, hierarchical cluster analysis, and the PLS –SEM methods, which will be introduced in the following subsections (Section 3.1 and Section 3.2), will be adopted for retrieving the problem based on the idea of visualization to analyze invisible phenomenon as a latent factor in text data. The proposed process consists of the following four steps (see as Figure 2): 

### 3.1. Text-Mining, Topic Model and LDA

Text-mining has become a popular topic across a wide range of fields such as public health, disaster management, and environmental management [71]. Text-mining techniques are used to extract knowledge to derive meaningful structural information from irregular data patterns or unstructured forms of data to provide meaningful information patterns in the shortest time period [72]. Thus, during the past years, text-mining methods have widely been adopted in numerous research fields such as computational linguistics, information retrieval, data mining, etc.

Topic modelling, a machine learning technique in the field of data mining, is the most popular analytic method for the text-mining of social media. Topic modelling is the process of learning, recognizing, and extracting high-level semantic topics across a corpus of unstructured text. The technique is used to clarify the structure of a group of documents by estimating the word-distribution that constitutes a topic based on the premise that each group of documents, which constitutes a corpus belongs to a specific topic [73]. Amongst the techniques, LDA is one of the most popular methods [74]. 

**Figure 3:**
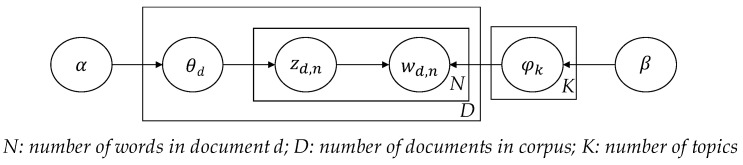
LDA graphic model. Source: adapted from [75].

LDA, a generative probabilistic model of a corpus, is an unsupervised machine learning technique that aims to identify the information in latent topics in a collection of large documents. The basic idea of LDA is that the documents are represented as random mixtures over latent topics, where a topic is characterized by a distribution over words. LDA treats each document as a vector of words. Each document is represented as a probability distribution over some topics, while each topic is represented as a probability distribution over a number of words. Figure 3 shows the graphic model of the LDA where:

*α* is the per-document topic distributions,
*β* is the per-topic word distribution,
$\theta_{d}$ is the topic distribution for document *d*,
$\varphi_{k}$ is the word distribution for topic *k*,
$Z_{d,n}$ is the topic for the *n*-th word in document *d*, and
$W_{d,n}$ is the specific word.

LDA defines the following generative process for each document in the collection: 1. For each document, pick a topic from its distribution over topics. 2. Sample a word from the distribution over the words associated with the chosen topic. 3. The process is repeated for all the words in the document. The generative process of LDA is as follows:

For each topic *i* in *(1, K):*
Choose per-corpus topic distribution $\varphi_{k}\sim Dir\left(\beta \right)$
For each document *i* in *(1, D):*
  Choose per-document topic proportion $\theta_{d}\sim Dir\left(\alpha \right)$
  For each word *j* in *(1, N):*
  Choose topic $Z_{d,n}\sim Multinomial\left(\theta_{d}\right)$ with $Z_{d,n}\in \left[1,K\right]$
  Choose word $W_{d,n}\sim Multinomial\left(\varphi_{k}\right)$ given $Z_{d,n}$

### 3.2. PLS-SEM

The SEM methods are widespread in marketing and management research while analyzing the cause–effect relations between latent constructs. The PLS-SEM is one of the SEM methods, which aims to maximize the explained variance of the dependent latent construct [76]. As with other SEM techniques (e.g., the linear structural relations, the LISREL), the PLS approach allows researchers to simultaneously assess the parameters of the measurement model and the coefficients of the structural path. The covariance-based SEM techniques, such as the LISREL and the EQS, use a maximum likelihood function to obtain estimators in models. Instead, the PLS-SEM uses a least-squares estimation procedure. PLS-SEM avoids many restrictive assumptions that underlie the covariance-based SEM techniques, such as multivariate normality and large sample size. PLS-SEM analyzes various relationships among several factors, i.e., latent and observed variables. A latent variable is an invisible concept for a target analysis. An observed variable is an observable item from a target analysis and is used to estimate a latent variable. These variables have relationships, such as causal and co-occurrence relationships. PLS-SEM was used as the research method in this work because the sample size may be non-normally distributed due to the nature of the data being retrieved from social media websites.

## 4. Social Media Mining and Theoretical Framework Confirmation Results

This section presents the process of social media mining and the process of confirming the theoretical framework being developed in Section 2.5. Based on the proposed hypotheses, we collected data from the largest anonymous social media platform in Taiwan, Dcard, to test the research model. With over four million registered members, averaging 15 million unique monthly visitors and 1.5 billion monthly views, Dcard is a wildly popular site where students and young people go to talk about life, work, politics and social issues. All the registered members are required to register the gender data and tag on each message. Thus, the website is very suitable for investigating gender differences in specific domains.

### 4.1. Data Crawling and Pre-Processing from Social Media

The messages were mined using Dcard API to retrieve posts related to users’ attitudes toward problems of air pollution in Taiwan. We used a simple keyword-based filter that identified one of four relevant terms (“air pollution”, “PM2.5”, “emission of air pollution”, and “air quality”) in Mandarin. All messages were collected in September 2020, but the messages were written as far back as 2016, when the first messages related to air pollution were posted on Dcard. The messages are from chat rooms about topics such as mood, chats, science, news, computers, communication and consumer electronics products, beauty, life, pets, online shopping, cars, and so on. The text preprocessing of removing punctuation, common “stop words”, and infrequent words, and performing Chinese word segmentation were carried out using a program we coded in Python 3.7 (https://www.python.org/downloads/release/python-370/; accessed on 7 June 2020). The data consists of 3700 messages collected in Dcard. After the data cleaning process of removing duplicates, errors, and messages unrelated to air pollution, the final sample consisted of 1043 messages, of which 60% were posted by the male population and 40% were posted by the female population.

Many researchers have proven that useful information and knowledge can be obtained from social media data [77]. Although social media mining can extract hidden information in the text, the social media content is usually affected by its typically noisy nature, such as abbreviations and/or irregular forms. A rigorous data quality assurance procedure is needed to reduce the noise of textual data and validity of data quality. To this end, we applied a four-step procedure in this research. The first step was to eliminate the short messages, which are unlikely to contain any mentions of air pollution. We calculated the average messages’ length, which was 482 words in this study, and removed the posts that were less than 25 words. Those posts being removed were confirmed again by the authors as useless for further analyses. The second step was to delete meaningless non-words such as the website URLs, emojis, and special characters [78]. The third step was to remove unsuitable keywords, such as stop words, by all the authors from the results of topic model execution, and iterate this step λ times (five times in this study) to assure suitable and reliable data quality [79]. The fourth step was to assess the validity and reliability of a measurement model in PLS-SEM. All the selected messages were reviewed by the authors to filter out irrelevant messages. 

### 4.2. Topic Extractions Using the LDA 

To retrieve topics embedded in the retrieved posts that were related to air pollution, we adopted LDA to analyze terms and topics from the entire corpus and to produce a topic model. The topic-specific word distributions typically give a high probability to words that tend to occur together in documents. Each topic can therefore be interpreted as a topically or semantically coherent group of words. Clusters of topic-specific words were distributions typically giving a high probability to indicate a topic; more effective mixtures of topics were derived for the probability distributions. The 1043 documents were converted into preprocessing documents and future computations by the topic modeling module. The LDA model parameters were estimated after 1000 iterations of Gibbs sampling, using 12 topics for our dataset. Based on the LDA, 12 topics with coherent groups of keywords, which can clearly describe the associated meanings, were defined by four environmental experts. The four environmental background experts included two professors working in environmental education and environmental economics and two senior researchers working in environmental disaster management for over 10 years. The 12 topics are policy ambiguity (*t*_1_), wind power generation policy (*t*_2_), fuel (*t*_3_), masks (*t*_4_), electronic cigarettes (e-cigarettes) ($t_{5}$), coal-fired power generation (*t*_6_), refuse combustion ($t_{7}$), climate change (*t*_8_), smoking (*t*_9_), allergies and health (*t*_10_), power generation (*t*_11_), and air purifiers (*t*_12_).

### 4.3. Topic Clustering Using the Hierarchical Cluster Aanalysis 

In order to eliminate the correlations and retain the variability of these topics, hierarchical cluster analysis was adopted to build a tree diagram for topic grouping. Hierarchical cluster analysis was used to convert a set of possibly correlated observations into a set of linearly uncorrelated components. Based on the results of clustering, the topics were classified into four themes as identified in the theoretical framework: egoistic concerns, altruistic concerns, biosphere concerns, and adaptation strategies (see Table 1). 

### 4.4. Path Model Construction Using PLS-SEM 

After classifying each topic as an observed variable into the constructs belonging to the theoretical framework of the value-belief-norm theory (refer Figure 1), we further confirmed the causal relationships using the PLS-SEM. To achieve our purpose, we normalized the distribution of topic numbers, which is a parameter of the LDA (refer Figure 3). Here, each document within the corpora was represented as a probability distribution over topics, while each topic was represented as a probability distribution over a number of words. In this study, we regard four themes as latent variables and 12 topics as observed variables. The two-step process, including (1) analysis of the measurement model and (2) the structural model, was adopted to confirm the theoretical framework by using the PLS–SEM method [80]. First, the measurement model was adopted to test the relationships between constructs and their indicators. Then, the causal relationships amongst the constructs, i.e., the hypotheses of the theoretical model, were confirmed.

#### 4.4.1. Measurement Model 

The measurement model was assessed in terms of individual item reliability, construct reliability, convergent validity and discriminant validity. Individual item reliability was analyzed through the factor loadings. All items exceeded the cutoff value of 0.7, which suggests that the items are acceptable (refer Table A1). Cronbach’s alpha, Dijkstra-Henselaer’s rho coefficient, and composite reliability (CR) were used to evaluate construct reliability. While traditionally, CB-SEM models use Cronbach’s alpha and CR to evaluate the construct internal consistency reliability, CR provides a more appropriate measure of internal consistency reliability for PLS-SEM exploratory models. This is because the Cronbach’s α test is sensitive to the number of items that may generate underestimated results in the reliability analysis for PLS-SEM exploratory models. Therefore, we apply CR to evaluate the internal consistency reliability of constructs. All CR values (see Table A1 in the Appendix) are greater than 0.600, which suggests an acceptable reliability according to Hair Jr. et al. [81]. 

In addition, convergent validity was assessed by examining the average variance extracted (AVE), which provides the sum of variance that a construct gains from its items in relation to the amount of the variance accounted for by measurement error. The analytic results in Table A1 also demonstrate good convergent validity and reliability because the AVE values range from 0.592 to 0.682, which is larger than the threshold value of 0.500 recommended by Bagozzi and Yi [82]. Hence, the convergent validity of the measurement model is acceptable. 

This study uses two common approaches, the Fornell–Larcker criterion and the cross-loading criterion [83], to examine discriminant validity. The Fornell–Larcker criterion was examined by comparing the square root of AVE with the correlations between the focal construct and all the other constructs. In this dataset, all the variables can fulfill the Fornell–Larcker criterion because the square roots of each AVE are higher than the correlations between the other latent variables (see Table A2), which demonstrates adequate discriminant validity for all dimensions. Moreover, discriminant validity can be further verified by using the cross-loadings assessment proposed by Chin [84]. Table A3 demonstrates that each of the factor loadings (digits in bold) is greater than all of the cross-loadings.

#### 4.4.2. Structural Model

After the verification of the measurement model, the structural model can be confirmed. The analytic findings are presented in Table 2 and Figure 2. The significance levels of the path coefficients are derived based on applying the bootstrap resampling method with 5000 subsamples. The hypothesis testing results are determined by inspecting these *p*-values in terms of the empirical study results. The variance inflation factors (VIFs), which evaluate every set of predictors for possible collinearity, are also presented in Table 2. The VIF of the indicators should be computed to identify the multi-collinearity problem [85]. As shown in Table 2, all the VIF scores ranged from 1.000 to 2.279, which are lower than the maximum level (5) of VIF [86]. Thus, the collinearity is acceptable according to Shiau and Chau [87]. 

Moreover, this study assessed the quality of the evaluation criteria by calculating the cross-validated predictive relevance of the model based on the value of the cross-validated predictive relevance of the model, based on the value of Stone-Geisser’s Q^2^, in which ranged from 0.252 to 0.305 (refer Table 2). All the values of Stone-Geisser’s Q^2^ are above zero, which suggests a good fit in model prediction. In addition, the model fit was tested based on the standardized root mean square residual (SRMR) to evaluate the difference between the observed correlation, and the model implied a correlation matrix. SRMR is the square root of the sum of the squared differences between the model-implied and the empirical correlation matrices. The value of SRMR is 0.082 in this research, which is less than the maximum level of 0.100 [88]. This result (see Table 2) indicates that the overall model fit is acceptable. 

#### 4.4.3. Hypothesis Test Results 

**Figure 4:**
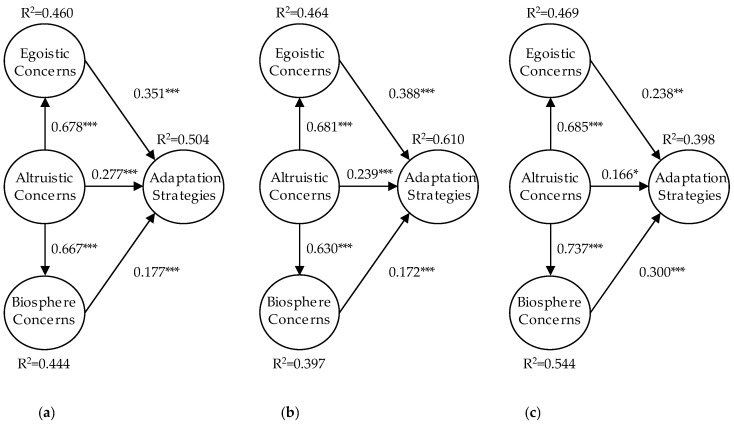
Path analysis results for posts by (**a**) both genders, (**b**) men, and (**c**) women. Notes: *: *p* < 0.050; **: *p* < 0.010; ***: *p* < 0.001.

Based on the analytic results shown in Table 3 and Figure 4a, we tested the five hypotheses proposed in Section 2.5 using PLS-SEM. Figure 2 illustrates the *R*^2^ and the path coefficients of the proposed research model. The estimation of the path coefficient indicates the strength of the relationship between the dependent and independent variables. The value of $R^{2}$ represents the amount of variance explained by the independent variables. Additionally, the value of *R*^2^ and the path coefficients together indicate how well the data supports the hypothetical model. The proposed model explains 50.400% of the variance for the factor of adaptation strategies, which is generally considered a moderate effect. The variances of EC and BC are under 50.400%, which could be considered a weak effect.

For the results of hypothesis testing, H1 was supported: altruistic concerns were significantly related to egoistic concerns (*β* = 0.678, *p* = 0.000). For H2, altruistic concerns were significantly related to biospheric concerns (*β* = 0.667, *p* = 0.000). For H3, egoistic concerns were significantly related to adaptation strategies (*β* = 0.351, *p* = 0.000). H4 predicted an effect of altruistic concerns on adaptation strategies, and this effect was also significant (*β* = 0.277, *p* = 0.000). Finally, for H5, biospheric concerns were significantly related to adaptation strategies (*β* = 0.177, *p* = 0.000). Thus, the PLS-SEM results fully supported H1, H2, H3, H4, and H5. The hypothesis testing results are presented in Table 3.

Regarding the implications of the research results, the direct, indirect, and total relationships with the criteria within each dimension are shown in Figure 2 and Table 4. Here, the aspect of egoistic concerns (*β* = 0.351) appears to be the most correlated aspect of AS while the aspect of altruistic concerns (*β* = 0.277) is the second. Furthermore, the correlations between altruistic concerns and adaptation strategies can be derived through three paths: AC → EC → AS (*β* = 0.678 × 0.351 = 0.238), AC → BC → AS (*β* = 0.667 × 0.177 = 0.118), and AC → AS (β = 0.277). We tested the role of egoistic concerns and biospheric concerns in mediating the relationship between altruistic concerns and adaptation strategies by using the variance accounted for (VAF). The VAF for egoistic concerns was 0.512% ((0.678 × 0.351)/(0.678 × 0.351 + 0.277) × 100% = 0.512%). The VAF of biospheric concerns is 0.342% ((0.667 × 0.177)/(0.667 × 0.177 + 0.277) × 100%). Both egoistic concerns and biospheric concerns are generally considered as partial mediation effects according to Hair Jr. et al. [89].

#### 4.4.4. Multi-Group Analysis 

To better understand the role of gender differences, we additionally examined the moderating role of gender in the relationships included in the theoretical model. We used multi-group analysis in PLS-SEM to test whether the five hypothesized relationships were moderated by gender. Based on the analytic results of the multi-group analyses, the relationships predicted by egoistic concerns and adaptation strategies (H3) and altruistic concerns and adaptation strategies (H4) were moderated by gender (see Table 5). In Table 6, the priorities of men and women regarding which environmental concerns were most important is different. The environmental concerns of men are prioritized as (1) egoistic concern to adaptation strategies (EC → AS, β = 0.388), (2) altruistic concern to adaptation strategies (AC → AS, β = 0.239), and (3) biosphere concern to adaptation strategies (BC → AS, β = 0.172). In contrast, the environmental concerns of women are prioritized as (1) biosphere concern to adaptation strategies (BC → AS, β = 0.300), (2) egoistic concern to adaptation strategies (EC → AS, β = 0.238), and (3) altruistic concern to adaptation strategies (AC → AS, β = 0.166), respectively. 

## 5. Discussion

In this study, we attempted to assess individuals’ concerns about air pollution and their adaptation behaviors; furthermore, the relationships between individuals’ concerns and adaptation strategies were assessed. Based on the analytic results, we would like to discuss the topics of most concern among Taiwanese, the relationships between environmental concerns and adaption strategies, impacts of environmental concerns on adaptation strategies, and moderations of gender in the relationship of altruistic and adaptation strategies in the following sub-sections: Section 5.1, Section 5.2, Section 5.3 and Section 5.4. 

### 5.1. The Topics of Most Concern for Taiwanese

Based on the results of topic modeling (refer Table 1), twelve topics have been identified. The inconsistencies with prior research will be discussed in this sub-section. Wang et al. [21] analyzed the contents of a mainland Chinese social media site that were related to air quality and air pollution. Based on that work, people’s major concerns regarding air pollution were prioritized as follows: (1) individual reactive behavior, (2) health concerns, and (3) requested actions to improve air quality such as reducing carbon emissions [21]. In comparison with the analytic results by Wang et al. [21], our study found that Taiwanese concerns were more about requesting improved actions such as reducing air pollutant emissions than individual reactive behavior and health risks. 

In addition, according to Wang et al. [21], the most widely adopted individual reactive behaviors of Chinese people are facemask-wearing, clothes-washing and staying indoors. However, Taiwanese concerns are primarily about masks, air purifiers and e-cigarettes. The concerns of Taiwanese people about e-cigarettes (*t*_5_) could be attributed to the emerging trend of adopting e-cigarettes by teenagers. After enacting the Tobacco Hazards Prevention Act [90], the Taiwanese government now prohibits smoking in all workplaces and other public places, and requires warnings to appear prominently on cigarette packages that encourage people to stop smoking. However, the diffusion rates of e-cigarettes from middle school to high school students have increased rapidly. Thus, the problems of e-cigarettes on polluting indoor air quality, chemical compositions, e-cigarette aerosols, and associated cardiovascular were broadly discussed on social media sites. 

From the perspective of the emission-sources of air pollution, the major sources of outdoor air pollution include cooking and heating, vehicles, power generation, agriculture/waste incineration, and industry [6]. Lam et al. [91] found that the major stakeholders (e.g., governments, the environmental groups, and the news media) are mainly concerned about the emissions of pollutants and the control of end-of-pipe pollution in Hong Kong. The work by Lam et al. [91] indicated that the public was especially focused on the emissions from power plants and vehicles. According to the results of our analyses (refer to Table 1), five topics are related to energy: policy ambiguity (*t*_1_), wind power generation policy (*t*_2_), coal-fired power generation (*t*_6_), refuse combustion (*t*_7_), and power generation (*t*_11_). These results may be attributed to the Taiwan’s energy dilemma between anti-nuclear energy and anti-air pollution from fossil fuel energy. 

According to the Energy Statistics of Taiwan [92], about 93% of Taiwanese energy consumption was from imported fossil fuels. Taiwan has been facing an energy policy dilemma due to the desirability of multiple goals: achieving a nuclear-free homeland, reducing air pollution from fossil fuels, and restricting the receiving capacity of liquefied natural gas terminals. Some people are against nuclear power because of the public anxiety from the Japan Fukushima Daiichi nuclear disaster in 2011 and problems with storing nuclear waste. Anti-nuclear activists advocate turning Taiwan into a nuclear-free country by 2025. Some people are against the coal-fired power generation plants because of the rise in air pollution causing 6000 air-pollution related deaths per year in Taiwan [93]. Anti-air pollution activists promote the view that nuclear energy could potentially provide an alternative form of power generation to decrease the use of coal-fired thermal power. The debates regarding energy policy have led to political and legal limbo regarding air pollution in the 2018 Taiwan referendum. Our research results are in line with the realistic social situation. The results are also consistent with previous studies showing that air pollution is closely linked with energy selection. For example, Heinrichs [94] examined the phasing out of coal-fired power plants in Germany from social, macro-economic, and technical perspectives. Based on the work on social perspective, the prevailing positive attitude towards a coal phase-out in German society is also reflected in the willingness to accept the consequences of the transformation process such as being willing to accept wind turbines near their homes in order to reduce the emission of air pollution. Severnini [95] exploits the shutdown of nuclear facilities in the Tennessee Valley after the Three Mile Island accident in 1979 to estimate its direct impact on coal-fired power generation in the context of particle pollution. The results show that the total suspended particulate concentration increased significantly as a result of the substantial increase in coal-fired power generation after the nuclear shutdown.

### 5.2. The Relationship among Egoistic, Altruistic, and Biosphere Concerns

As shown in Figure 4a, altruistic concern is positively related to egoistic concern. Thus, H1 is supported. Altruistic concern is also positively related to biosphere concern, which supports H2. The results for H1 and H2 are directly relevant to two suggestions in the literature. The value-belief-norm theory being proposed by Stern et al. [9] suggests that egoistic, altruistic, and biosphere concerns are mutually correlated. They also suggested that altruistic concern and biospheric concern together make up a general-altruistic concern; the structure derived from that notion is referred to here as the self and non-self structure. Snelgar et al. [9] found positive correlations between the concern scores: for altruistic and egoistic concerns, it was moderately high; for altruistic and biospheric concerns, it was moderate. In addition, the finding that altruistic concerns are positively correlated with egoistic concerns in our study supports the inclusion model for environmental concern. The inclusion model for environmental concern, proposed by De Dominicis et al. [33], suggests that egoistic concerns are included within altruism. That is, to be concerned for the biosphere and for all living things (altruism) does not happen in the absence of self-interest (egoistic). 

### 5.3. Impacts of Environmental Concerns on Adaptation Strategies

As shown in Figure 3, the egoistic concern, altruistic concern, and biosphere concern are positively associated with adaptation strategies. The analytic results are consistent with the hypotheses H3, H4, and H5. Overall, the three environmental concerns explain 50.4% of the variance of the adaptation strategies (*R*^2^ = 0.504). The positive correlations among altruistic and biospheric concerns as well as adaptation strategies are consistent with previous research by Helm et al. [51], which suggested that altruistic and biosphere environmental concerns positively influence the psychological adaptation to climate change. 

According to our results, egoistic concern is positively related with adaptation strategies. The relationship between egoistic concerns and pro-environmental behavior has been discussed by numerous scholars. Schultz et al. [29] argue that individuals oriented toward self-interest are likely to engage in pro-environmental behaviors in a self-enhancing situational value frame rather than in a self-transcendent one, whereas altruistic-oriented individuals are likely to engage in pro-environmental behaviors both in a self-enhancing and self-transcendent situational value frame. Self-enhancement refers to concern for self-interest, while the self-transcendence value refers to concern for the welfare and interests of others. It implies that an egoistic self-interested orientation tends to be negatively associated with environmental concern and action. People with a strong egoistic value orientation will especially consider the costs and benefits of pro-environmental behavior for them personally. Acting on egoistic values implies not behaving pro-environmentally because the personal costs associated with the pro-environmental behavior outweigh the personal benefits [96]. In addition, Crompton and Kasser [97] suggested that environmental campaigns should avoid promoting selfish motivations such as reducing carbon emissions to save money; rather, they should increase awareness of the inherent value of nature and empathy for nonhuman animals. However, we have shown that egoistic concerns can also lead to pro-environmental behavior. The dual motives for risk perception may provide an explanation. Self-interest (egoistic) may become positively related to environmental attitudes in situations where a serious environmental problem is directly and perceptibly threatening to the individual [29]. The seriousness of environmental problems may not reach a sufficiently malignant level to activate self-interest. Song and Kim [98] also proposed that the public may have dual motives of benefitting others and themselves when they have an awareness of the harmful environmental consequences, then feel egoistic and altruistic concerns jointly, correspondingly. In our research, people in Taiwan have a strong perception that air pollution is the most serious of all environmental problems, with negative consequences for their own and others’ health and safety. For example, in 2018, Taiwan passed referendums on three topics related to air pollution: opposition to thermal power plants, opposition to coal-fired power generation plants, and abandoning turning Taiwan into a nuclear-free homeland in 2025 [99]. The turnout for the referendum was 50 to 55 percent. In this paper, we present evidence that egoistic, altruism and biosphere concerns can provide pathways to pro-environmental behavior.

According to the analytic results (refer Figure 4a), the path strength of egoistic concern to adaptation strategies (EC → AS, *β* = 0.351) is stronger than altruistic concern to adaptation strategies (AC → AS, *β* = 0.277) and biosphere concern to adaptation strategies (BC → AS, *β* = 0.177). The finding indicates that egoistic concern is strongly related to adaptation strategies. The result is contrary to those of prior research on climate change. Steg et al. [57] and Helm et al. [51] suggested that the biosphere value is a stronger predictor of pro-environmental beliefs, attitudes, norms, and actions than either egoistic or altruistic environmental concerns for climate change-related issues. In our research, individuals with strong egoistic concerns are most likely to be concerned about air pollution adaptation strategies. The perception of risk for different environmental problems may provide the explanation. According to Bickerstaff et al. [100], public understandings of the risks of air pollution draw upon evidence from: (1) the physical experience of pollution, (2) fewer substantial forms of evidence, (3) information relayed through social networks, and (4) observed or reported impacts on the wider environment. In the context of Taiwanese concerns related to air pollution, public egoistic concerns are based on physical experience of pollution such as second-hand smoke or e-cigarettes. So, individuals are likely to find solutions to act on. The major altruistic concerns are related to public administration issues based on news or social media information such as coal-fired power generation. Individuals have divergent considerations and hesitate to decide on adoption strategies. 

Our findings indicate that egoistic concern regarding air pollution is more related to adaptation strategies than altruistic or biosphere concerns are to adaptive strategies. This result is also in line with Bain et al. [101], who argued that appealing to societal and self-interested benefits could be more effective than focusing on ecological outcomes or altruistic environmental issues, and motivate behavioral change among climate change deniers.

### 5.4. Moderations of Gender in the Relationship of Altruistic and Adaptation Strategies

Besides the effects of different environmental concern on the adaptation strategies, the research further emphasized that gender is a significant moderating variable in the relationship of egoistic concerns, altruistic concerns, and adaptation strategies. According to the results of our analysis (see Table 5), the path coefficients from egoistic concern to adaptation strategies (EC → AS) and altruistic concern to adaptation strategies (AC → AS) for men are significantly greater than the corresponding path coefficients for women; hence, the relationships hypothesized by H3 and H4 were moderated by gender. It means that, when there is a fit between egoistic concern or altruistic concern and pro-environmental behavior, men are more likely to link concerns to actions than women. The results are aligned with those of the study by Swami et al. [66], finding that only egoistic and altruistic concerns were significantly associated with sex, whereas biosphere concern was not significantly associated with sex. 

However, our results differ from those of previous studies that indicated that women tend to show higher levels of concern about environmental problems than men do [60,66]. Vicente-Molina et al. [68] confirmed that women are more altruistically motivated and behave more responsibly towards the environment. Liao et al. [48] found that women tend to have a greater knowledge of air pollution than men. There could be two possible reasons for this unexpected result. One explanation is based on the system justification perspective, which suggests that people’s attitudes of desire not only depend on themselves but also on the overarching social structure to which they are obligated. In this study, men chronically engage in more system justification than women do, which partially explains men’s greater willingness to acknowledge ecological problems and risks and to engage in actions that are beneficial for the environment [102]. Another explanation may be related to gender differences in social media use. Women are more avid users of social media [103]. In our research, all topics reflecting egoistic and altruistic motives are brought up by men more than women. In the education context, this implies that educators can emphasize egoistic and altruistic concern topics in promoting an adaption strategy to air pollution to increase female citizens’ concern.

In addition, our study shows that the priorities of men and women regarding which environmental concerns were most important are different, as shown in Figure 4b,c. These findings suggest that the gender differences exist in the relationship between air pollution concerns and action intentions. The results may be explained by the social construct expectation [104]. Men are socialized to be protectors of and providers for the family and may portray environmental pollution as a necessary tradeoff for growth. Men are more likely to link egoistic and altruistic concerns to actions. In contrast, women are more concerned about biosphere environmental problems because women are socialized to be family nurturers and caregivers for children and have greater social responsibility and empathy towards others. Women are more likely to link biosphere concerns to actions [105]. 

## 6. Conclusions

Air pollution is one of the highest environmental risks to health and is regarded as an urgent issue in the world. There is a growing awareness that environment quality has been greatly threatened by air pollution. Various studies have explored the relationship between air pollution, health, and the environment. A key, but generally absent, element of successful environment policy is a high level of awareness, positive attitudes, and support from citizens. Such policy requirements are in citizens’ interest. Social media mining has been shown to be a useful tool for public-opinion extraction and analyses of air pollution. Compared with traditional questionnaire-based surveys, social media mining can retrieve knowledge from large unstructured data from booming social media sources. These social media provide instant public responses and capture long-term data with low cost. Environmental and social scientists have realized the potential of social media as a source of data and have conducted numerous studies regarding public opinions. These studies have examined messages on social media regarding air pollution. However, how to successfully exploit big data being retrieved from social media and gain valuable insights into public pro-environmental behaviors still needs further investigation. In pursuit of this aim, this study has not only analyzed the content of social media but has also transformed empirical validations of theoretical concepts in environmental research. 

The contributions of this research are twofold. First, the combination of theoretical and practical considerations in this research has important implications for both academics and policy planners in gaining a better understanding of public concerns related to air pollution. In the past, many scholars have emphasized the importance of studying the relationships between human values and environmental behaviors. Various scholars argue that egoistic, altruistic, and biospheric value orientations are important for understanding environmental beliefs and behaviors [52]. Values may also affect the extent to which people are aware of environmental problems being associated with their behaviors. Awareness of consequences will increase if important environmental values are threatened, and people may adjust their behavior in order to reduce this threat. This research examines the different effects of egoistic, altruistic, and biospheric concerns regarding air pollution on adaptation strategies by using new hybrid social media mining and the PLS-SEM technique. The results provide support for the generalization of the egoistic, altruistic, and biospheric interactions and their contributions to adaptation strategies. Second, this paper proposes a novel hybrid data analysis approach combining two kinds of research models: social media mining based on big data analysis and the PLS-SEM based on multivariate analysis techniques. We first used the LDA to analyze un-structured text data retrieved from social media sites. Then, we clustered the public concerns from LDA results into the theoretical framework based on the value-belief-norm theory. Finally, we confirmed the causal model using the PLS-SEM. The novel analytic framework provides researchers and policy-makers with a new approach to social media data analyses. 

This research has several practical applications that are worthy of future study. First, this study suggests that egoistic concerns are a stronger predictor of adaptation strategies for air pollution rather than altruistic or biospheric concerns. Additional scientific questions may arise, leading to a greater understanding of the joint effects of egoistic and altruistic concerns. Second, this study suggests that the moderating effect of gender difference exists in the paths from egoistic concerns to adaptation strategies and the path from altruistic concerns to adaptation strategies. Gender is less evident as a part of current air quality policy in practice, although women have been identified as vulnerable yet crucial players in leading their neighborhoods towards a safer environment. Seeing air pollution through women’s eyes reflects women’s specific needs and interests in air pollution. More research regarding the potential association between air pollution and gender-related differences would be worthy of further development and exploration.

This research has two limitations. First, the present study uses the social media site Dcard as the data source, but Dcard users may not be representative of the population at large, as social media sites such as Dcard may be more widely adopted by the younger population. Second, most social media posts may be from subgroups who are likely to suffer from problems of representativeness [106]. Most of the social media posts examined in this study were related to personal or social interests. The posts in general and by women in particular that were related to biosphere concerns were relatively rare. For future studies, linking social data with other trusted information sources—for example, survey data or air pollution monitoring data—may be helpful for confirming theoretical frameworks from other aspects. 

## Figures and Tables

**Table 1:** Identified topics and topic clustering.

Latent Variables	Item Code	Item Name	Count (Men/Women)	Top Five Keywords Belonging to Each Topic
Egoistic Concerns	*t*_3_	Fuel	55 (36/19)	U.S., Taiwan, natural, fuel, smoking forbidden
	*t*_4_	Mask	146 (99/47)	air, air pollution, air quality, mask, research
	*t*_5_	E-cigarette	50 (36/14)	e-cigarette, tobacco, Taiwan, harm reduction, cigarette
	*t*_9_	Smoking	140 (83/57)	smokes, cigarette smoke, cigarette butts, smells, school
Altruistic Concerns	*t*_6_	Coal-fired power generation	61 (42/19)	shen’ao power plant, air poolution, govermenal, EPA^(^*^)^, coal burning
	*t*_7_	Refuse combustion	62 (39/23)	air, garbage, earth, burning, joss paper
	*t*_11_	Power generation	68 (50/18)	tai-power, power plant, power unit, generator set, gas
Biophere Concerns	*t*_1_	Policy ambiguity	30 (26/4)	plebiscite, green with nuclear, nuclear, gavernment, cosignatory
	*t*_8_	Climate change	83 (63/20)	climate, energy, global, climate change, renewable energy
Adaptation Strategies	*t*_2_	Wind power generation policy	64 (39/25)	Taiwan, wind power, offshore wind power, polar bear,
	*t*_10_	Allergy and healthy	165 (56/109)	allergy, nose, dortor, feel
	*t*_12_	Air purifier products	119 (52/67)	air purifier, allergy, recommad, air filter

Remark: * EPA is the abbreviation of the Environment Protection Agency, Taiwan.

**Table 2:** Significance testing results of the structural model path coefficients.

Hypothesis	Sample Mean (M)	Std. Deviation (STDEV)	Path Coefficients (*β*)	*t* Statistics	*p* Values	VIF
H1 (AC→EC)	0.680	0.018	0.678	37.433	0.000	1.000
H2 (AC→BC)	0.668	0.020	0.667	32.721	0.000	1.000
H3 (EC→AS)	0.351	0.037	0.351	9.352	0.000	2.203
H4 (AC→AS)	0.277	0.041	0.277	6.776	0.000	2.279
H5 (BC→AS)	0.178	0.039	0.177	4.517	0.000	1.966

Remark: $Q_{EC}^{2}=0.252;\;Q_{BC}^{2}=0.282;\;Q_{AC}^{2}=0.305;SRMR=0.082.$

**Table 3:** Hypothesis testing results.

Hypotheses	Results
H1 (AC → EC)	Supported
H2 (AC → BC)	Supported
H3 (EC → AS)	Supported
H4 (AC → AS)	Supported
H5 (BC → AS)	Supported

**Table 4:** Direct, indirect, and total effects.

Relationships	Direct	Indirect	Total
H1 (AC → EC)	0.678	N.A.	0.678
H2 (AC → BC)	0.667	N.A.	0.667
H3 (EC → AS)	0.351	N.A.	0.351
H4 (AC → AS)	0.277	0.356	0.277
H5 (BC → AS)	0.177	N.A.	0.177

Remark: N.A. means not applicable.

**Table 5:** Multi-group comparison test results.

Relationships	Path Coefficients-Diff (Men–Women)	*p*-Value (Men–Women)
H6a (AC → EC)	0.004	0.540
H6b (AC → BC)	0.107	0.997
H6c (EC → AS)	0.150	0.044 *
H6d (AC → AS)	0.163	0.036 *
H6e (BC → AS)	0.128	0.919

Notes: *: *p* < 0.050.

**Table 6:** Gender differences of environmental concerns.

Rank	Men	Women
1	EC → AS	BC → AS
2	AC → AS	EC → AS
3	BC → AS	AC → AS

## Data Availability

The data set generated in this study are not publicly available due to the service-term restrictions of the social media platform.

## Appendix A

**Table A1:** Measurement validation.

Latent Variables	Items	Out Loadings	Cronbach’s Alpha	Dijkstra-Henseler’s Rho	CR	AVE
Egoistic Concerns			0.767	0.779	0.852	0.592
	*t*_3_	0.813				
	*t*_4_	0.651				
	*t*_5_	0.768				
	$t_{9}$	0.833				
Altruistic Concerns			0.698	0.706	0.832	0.623
	*t*_6_	0.813				
	*t*_7_	0.821				
	*t*_11_	0.732				
Biosphere Concerns			0.557	0.660	0.809	0.682
	*t*_1_	0.914				
	*t*_8_	0.726				
Adaptation Strategies			0.731	0.733	0.8432	0.623
	*t*_2_	0.816				
	*t*_10_	0.793				
	*t*_12_	0.809				

**Table A2:** Discriminant validity-Fornell–Larcker criterion.

Latent Variables	EC	AC	BC	AS
EC	**0.769**			
AC	0.678	**0.790**		
BC	0.611	0.667	**0.826**	
AS	0.647	0.633	0.576	**0.806**

Note: The square roots of AVEs are shown diagonally in bold.

**Table A3:** Discriminant validity–Cross-loading Criterion.

	Variables	EC	AC	BC	AS
Topics					
*t*_3_		**0.813**	0.582	0.522	0.552
*t*_4_		**0.651**	0.452	0.434	0.408
*t*_5_		**0.768**	0.523	0.464	0.474
*t*_9_		**0.833**	0.521	0.459	0.543
*t*_6_		0.555	**0.813**	0.544	0.509
*t*_7_		0.584	**0.821**	0.547	0.546
*t*_11_		0.459	**0.732**	0.485	0.438
*t*_1_		0.609	0.653	**0.914**	0.587
*t*_8_		0.359	0.410	**0.726**	0.317
*t*_2_		0.538	0.565	0.512	**0.816**
*t*_10_		0.528	0.475	0.439	**0.793**
*t*_12_		0.496	0.484	0.436	**0.809**

Note: Bold values are loadings for the items which are above the recommended value, 0.7. An items’ loading on its variable is higher than all of its cross-loadings with other variables.
